# Supplementary material for: Baseline Levels of Vitamin D in a Healthy Population from a Region with High Solar Irradiation
Source: Nutrients. 2021 May 13;13(5):1647. doi: 10.3390/nu13051647 (PMC8152961; doi:10.3390/nu13051647)
Supplement: Supplementary file 1 [file nutrients-13-01647-s001.zip › nutrients-1194170-supplementary.pdf]

Baseline Levels of Vitamin D in a Healthy Population from a Region with High Solar Irradiation

Alicia García-Dorta [1], Lillian Medina-Vega [2], Jacobo Javier Villacampa-Jiménez [2], Marta Hernández-Díaz [1], Sagrario Bustabad-Reyes [1], Enrique González-Dávila [3] and Federico Díaz-González [1,4,*]

Supplementary Materials:

Table S1. Distribution of the study population by place of residence, gender, and age.

	Rural		Urban		Total		Total
**Age (years)**	Male	Female	Male	Female	Male	Female	
20-44	41 (4%)	37 (4%)	235 (25%)	224 (23%)	276 (29%)	262 (27%)	537 (57%)
45-64	22 (2%)	20 (2%)	113 (12%)	112 (12%)	135 (14%)	132 (14%)	266 (28%)
≥65	13 (1%)	15 (1%)	51 (5%)	66 (7%)	64 (7%)	82 (8%)	146 (15%)
Subtotal	76 (8%)	73 (8%)	399 (42%)	402 (42%)	474 (50%)	475 (50%)	949 (100%)
Total	148 (16%)		801 (84%)		949 (100%)		

Data are expressed as n (%)

Table S2. Means (μ) and standard deviations (σ) of log-transformed VitD levels.

		Age (years)		
**Sex**		20 – 44	45 - 64	≥ 65
Male	μ	3.3611– 0.01615·(BMI–25.89)*	3.2538	3.2254
	σ	0.31875	0.34421	0.30959
Female	μ	3.2654 –0.01698·(BMI–24.92)*	3.1392	2.9873
	σ	0.38482	0.35410	0.34756

BMI: Body Mass Index

Table S3. Difference between percentiles obtained from sample data and those obtained by applying the condition of normality on the log transformation.

	Male (age, years)			Female (age, years)			Sex		Age group			Total
	20-44	45-64	≥ 65	20-44	45-64	≥ 65	Male	Female	20-44	45-64	≥ 65	Total
P_{10}	0.18	-0.55	0.45	-0.71	0.36	-0.20	0.13	-0.28	-0.25	-0.19	-0.09	-0.33
P_{20}	-0.12	-0.25	1.19	-0.66	-0.45	0.10	-0.08	-0.25	-0.50	-0.21	-0.07	-0.17
P_{30}	0.30	-0.14	-0.55	-0.54	-0.38	-0.84	0.01	-0.47	-0.67	-0.45	0.10	-0.39
P_{40}	0.79	0.41	-1.13	-0.34	-0.44	-0.43	0.23	-0.52	-0.06	-0.30	-0.12	-0.38
P_{50}	0.35	0.21	-0.89	-0.61	-1.14	0.16	0.07	-0.55	0.10	-0.40	-0.58	-0.31
P_{60}	0.35	0.25	-0.71	0.08	-0.95	0.29	-0.25	-0.15	-0.02	-0.10	-0.64	-0.02
P_{70}	0.15	-0.90	-0.85	0.40	-0.24	0.05	0.00	0.44	0.43	-0.45	-0.95	-0.10
P_{80}	-0.68	0.85	0.10	0.65	-0.75	-0.70	-0.05	0.79	-0.12	0.10	-0.61	0.72
P_{90}	0.51	-1.23	0.98	-0.88	2.13	-0.97	0.66	0.37	0.35	1.71	1.06	0.67
p	0.18	0.51	0.60	0.15	0.54	0.11	0.36	0.68	0.52	0.90	0.31	0.82
r_s	0.9986	0.9964	0.9914	0.9982	0.9909	0.9974	0.9995	0.9991	0.9995	0.9976	0.9961	0.9996

p: p-value comparing whether differences between the percentiles differ from zero; r_s = Spearman rho.
Cells are shaded green for differences in absolute values between sample and log-transformed data < 0.5; yellow for differences between 0.5 and 1, and red for differences > 1 in absolute values.
